# Insight into microRNAs-Mediated Communication between Liver and Brain: A Possible Approach for Understanding Acute Liver Failure?

**DOI:** 10.3390/ijms23010224

**Published:** 2021-12-25

**Authors:** Karolina Orzeł-Gajowik, Krzysztof Milewski, Magdalena Zielińska

**Affiliations:** Department of Neurotoxicology, Mossakowski Medical Research Institute, Polish Academy of Sciences, 5 Pawińskiego Str., 02-106 Warsaw, Poland; korzel@imdik.pan.pl (K.O.-G.); kmilewski@imdik.pan.pl (K.M.)

**Keywords:** acute liver failure, acetaminophen toxicity, circulating microRNA, hepatic encephalopathy, viral hepatitis

## Abstract

Acute liver failure (ALF) is a life-threatening consequence of hepatic function rapid loss without preexisting liver disease. ALF may result in a spectrum of neuropsychiatric symptoms that encompasses cognitive impairment, coma, and often death, collectively defined as acute hepatic encephalopathy. Micro RNAs are small non-coding RNAs that modulate gene expression and are extensively verified as biomarker candidates in various diseases. Our systematic literature review based on the last decade’s reports involving a total of 852 ALF patients, determined 205 altered circulating miRNAs, of which 25 miRNAs were altered in the blood, regardless of study design and methodology. Selected 25 miRNAs, emerging predominantly from the analyses of samples obtained from acetaminophen overdosed patients, represent the most promising biomarker candidates for a diagnostic panel for symptomatic ALF. We discussed the role of selected miRNAs in the context of tissue-specific origin and its possible regulatory role for molecular pathways involved in blood–brain barrier function. The defined several common pathways for 15 differently altered miRNAs were relevant to cellular community processes, indicating loss of intercellular, structural, and functional components, which may result in blood-brain barrier impairment and brain dysfunction. However, a causational relationship between circulating miRNAs differential expression, and particular clinical features of ALF, has to be demonstrated in a further study.

## 1. Background

### 1.1. Liver Disease a Global Burden

Liver disease does not usually cause unequivocally symptoms until it is advanced, and the liver is seriously damaged and dysfunctional. The liver comes in close contact with harmful substances and defends itself in two ways. First, it can regenerate by repairing or replacing injured tissue. Second, as it is composed of units with similar functions, thus, if one area is injured, other cells will perform the work of the injured section indefinitely or until the damage is repaired. Despite the existing, albeit, in theory, self-repairing and regenerative abilities, liver disease is an increasing global problem.

The burden of liver disease worldwide continues to grow. Each year, the liver disease accounts for about 2 million deaths, i.e., 1 million due to cirrhosis and 1 million due to viral hepatitis and hepatocellular carcinoma (HCC) [1,2]. Liver diseases account for a significant burden in terms of health and costs worldwide. This group of diseases encompasses epidemiologically diverse alcohol-related liver disease, non-alcoholic fatty liver disease, hepatitis caused by a viral infection, or exposure to drugs, hemochromatosis, an inherited disorder with a gradual build-up of iron usually around the liver, primary biliary cirrhosis, and liver tumors.

Epidemiological studies of liver disease limit caveats, such as heterogeneous population under study, incomplete ascertainment, lack of unified disease definitions, and method of assessment (laboratory tests, biopsy, non-invasive markers, imaging). The aforementioned caveats suggest that the global burden of both acute and chronic liver diseases is prevalent worldwide and expected to increase, causing significant mortality [3,4].

### 1.2. Acute Liver Failure

Acute liver failure (ALF), originally defined by Trey and Davidson in 1970 as fulminant liver failure, refers to a specific syndrome, characterized by loss of liver function that occurs rapidly—in hours or days, and by abnormality of liver blood tests, usually in a person who has no preexisting history of liver disease [5]. After two decades, the syndrome was redefined to take into account, i.e., etiology and prognosis [6]. Despite being less common than chronic liver disease, ALF is associated with a high mortality grade and approximately half of the cases progress and require liver transplantation so immediate diagnosis and management of that syndrome need to be applied [7,8]. A wide range of insults can create a clinical picture of ALF (Table 1). The most common causes of ALF are drugs overdose and hepatitis virus infection, causing about 70% of cases [9]. Moreover, prognosis suggests that many drug-induced ALF cases will grow and dominate as a major cause of ALF in the future decades.

Drug-induced liver injuries are clinically challenging due to their idiosyncratic (unpredictable) character arising from the enormous amount of commercially available drugs, herbs, and dietary supplements with hepatotoxic potential. Acetaminophen is the most common drug that indicates ALF. It is responsible for approximately 50% of cases of ALF in Western Europe and the United States, [10,11,12], while traditional complementary and dietary supplements dominate as the main causative factor of ALF in Asia [13]. A variety of clinical and pathological phenotypes with the current lack of specific biomarkers increases the uncertainty of liver disease diagnosis and output and requires highly careful exclusion of alternative diagnosis.

The second most frequent etiological cause of ALF is viral hepatitis infections affecting individuals in all geographic regions, but mostly in low and middle-income countries. In 2010, deaths from viral hepatitis accounted for 0.3 million per year, an increase of about 50% from 1990. There are five main types of hepatitis viruses, including type A, B, C, D, and E, of which, type B and C remain prevalent globally, albeit differ in the geographical distribution of disease prevalence.

In 2019, ~1.5 million (1.1–2.6 million) people newly infected with hepatitis B virus, and ~1.5 million (1.3–1.8 million) newly infected with hepatitis C virus [14], account for ~1.1 million cases of patient deaths per year globally. Viral hepatitis-related number of deaths is higher than the mortality caused by HIV (~680,000 deaths in 2020) or malaria (~400,000 deaths in 2020), not much less than deaths number caused by tuberculosis (~1.4 million in 2020), additionally exaggerated with the unprecedented COVID-19 pandemic, which disrupted health care globally [15,16]. ALF may also develop due to infections by other rare viruses, including herpes simplex virus, cytomegalovirus, Epstein–Barr virus [11], and parvoviruses. It is estimated that 10% of acute HBV infection progresses to chronic disease [17].

Other pathological conditions referred to as ALF causes are relatively rare but require mentioning as potentially life-threatened. Autoimmune hepatitis (AIH) accounts for 4 to 5% of ALF; it is typically presented in the third to sixth decades of life, more often in women than in men as a growing loss of tolerance to hepatocyte-specific autoantigens results in intensified autoimmune reaction [18]. Metabolic diseases, such as Wilson’s disease or acute fatty liver of pregnancy, besides their specific symptoms, may be accompanied by overwhelming infection (sepsis), which impairs blood flow to the liver, inducing ALF episodes. Additionally, a heatstroke combined with excessive physical activity can trigger ALF [19,20]. It is worth mentioning that despite extensive evaluation of patients’ medical history, blood tests, radiological studies, and liver biopsy, up to 20% of ALF cases remain indeterminate.

The frequent consequence of ALF is acute hepatic encephalopathy (A type of HE), a spectrum of neuropsychiatric symptoms ranging from subtle cognitive impairment to coma [21]. ALF-evoked HE develops as a result of accumulating toxic doses of liver metabolites, that may eventually pass from the circulation to the brain. Importantly, HE is often accompanied by massive systemic inflammation and severe intracranial hemodynamic alterations [22,23]. Cerebral edema defined as a pathological increase in total brain water leading to elevation of brain volume and intracranial pressure is one of the most serious complications of ALF, accompanied by impaired cerebral blood flow or even life-threatening herniation.

The scale of HE is often graded following the West Haven criteria (WHC) grade, the I–IV diagnostic scale range from minimal cognitive disturbances, sleep-wake cycle impairments, apathy, ataxia, up to coma and death [24]. Rapid cerebral edema diagnosis is essential to prevent irreversible brain damage. Unfortunately, diagnosis and scale determination of cerebral edema is difficult, despite patients displaying symptoms such as hypertension, a slow heart rate, abnormal reflexes, and loss of the reaction of the pupils to light. In addition, a head CT scan may reveal changes in the junction between gray and white brain matter, compression of the fluid-filled spaces, and loss of the brain surface folds (sulci). However, CT imaging and WHC evaluation of ALF patients are not a routine part of the liver disease diagnostic panel, especially in smaller health centers and in undeveloped countries.

Pathogenesis of HE is multifactorial, with several key factors that determine clinical manifestation, of which the dominant role of ammonia was repeatedly reported. However, lack of correlation between clinical picture and ammonia level is relatively frequent [25,26,27,28]. This clinically relevant fact often results from divergent factors affecting ammonia measurement, such as the lack of a unified methodology (venous or arterial blood for ammonia measurement, sample storage, temperature, pH- changes), and clinical determinants, including disease stage, renal function, sarcopenia, diet, etc. [28,29,30].

Significant but poorly explored is the blood–brain barrier (BBB) impairment during ALF. The BBB is a selective structure that, in normal conditions, guards the passage of neuro-toxic substances into the brain. The BBB composes the basement membrane, endothelial monolayer, pericytes, astrocytes end-feet, and at molecular level, many specialized proteins comprising tight, adherens junctions, and intracellular matrix. Alternation in each of these components can disrupt BBB integrity [31]. Ultrastructural analysis of the ALF patients’ cerebral cortex tissue confirmed structural BBB abnormalities [32], pointing to its potential role in the liver–brain crosstalk that may occur in ALF. However, details of BBB impairment, visible at both cellular and molecular levels, underlying this process are complex and are of continuous interest.

There is a need to identify more specific factors of ALF development to reduce patient death or the requirement for liver transplantation. The lack of ALF prognostic biomarkers makes the diagnostic appraisal of suspected cases strongly dependent on the interpretation of serum biochemistry and other routine laboratory tests and imaging evaluation to carefully rule out alternative causative factors of ALF. The liver enzymes activity panel: ALT, AST, GGT, ALP assisted with total bilirubin, and creatine is the standard laboratory screening procedure to define liver damage and liver dysfunction using blood samples. Of note, in this context, the AST measurement can be a reliable substitute for ALT in determining the injury when the latter is unavailable, whereas GGT is considered a less reliable ALP substitute [33,34].

### 1.3. Micro RNA

Micro RNAs (miRNAs) are small, approximately 18–23 nucleotides long, members of small non-coding RNAs which can negatively regulate gene expression at the mRNA level [35,36]. The first human miRNA was described in 2000 [37], and up to date, approximately 2700 human miRNAs have been described [open-source miRNA database “miRbase”, https://www.mirbase.org/, accessed on 22 November 2021]. It is estimated that approximately 50% of miRNAs are expressed from “non-coding” transcripts since the rest are located in the introns of protein-coding genes and are generally cotranscribed with their host genes and expressed separately [36].

In a general view, most miRNAs are transcribed by RNA polymerase II and then processed into a shorter hairpin-shaped precursor miRNA (pre-miRNA) by the class 2 nuclear RNase III Drosha complexed with DGCR8 [38]. Pre-miRNA is then exported from the nucleus into the cytoplasm by exportin 5 protein (Exp-5) [39]. Here, the pre-miRNAs proceed into 18–23 nucleotides long duplexes by RNase III enzyme Dicer-1. Finally, mature miRNAs (single-stranded) are incorporated into a stable multi-component protein complex called RISC which “guides” miRNAs into 3′ UTR sequence of the target mRNA [35]. The 3′ UTR blockage by partial base pairing with miRNA interrupts further translation. An alternative mechanism of action assumes that binding of miRNA leads to faster deadenylation of mRNA thereby decreasing mRNA stability and inducing their degradation [40].

miRNAs can act as negative regulators of gene expression at the posttranscriptional level by interacting with the 3′-untranslated region (3′-UTR) of the target mRNA sequence. Partial paring disturbs translation since complete pairing results in miRNA-mRNA complex degradation by nucleases [36]. In addition, miRNAs also exert their regulatory effects through some non-canonical ways: pri-miRNAs, the precursors of miRNAs, encode short peptides with regulatory functions; miRNAs are involved in the activation of Toll-like Receptors (TLRs); miRNAs upregulate the expression of specific proteins, and so forth [41]. Due to the complexity of these regulatory mechanisms, it is not surprising that miRNAs have been confirmed to be crucial players in many molecular pathways. The discovery of cell-free miRNAs in various body fluids, such as plasma, serum, saliva, urine, cerebrospinal fluid, bronchial lavage, suggests that miRNAs might act as signaling molecules outside the cell and may serve as suitable biomarkers that may support diagnosis and monitoring of treatment response for a variety of diseases [42,43,44]. MiRNAs expression profiles in body fluids present by definition a specific pattern that may exclude passive release from the injured cells and suggest its more selective release [45,46]. Furthermore, there is growing evidence that miRNAs can act as potential biomarkers that may be sensitive and specific for early diagnosis, prognosis, and therapeutic interventions, especially through their stability and easy detection in plasma or serum.

## 2. Study Concept and Data Analysis Strategy

**Figure 1:**
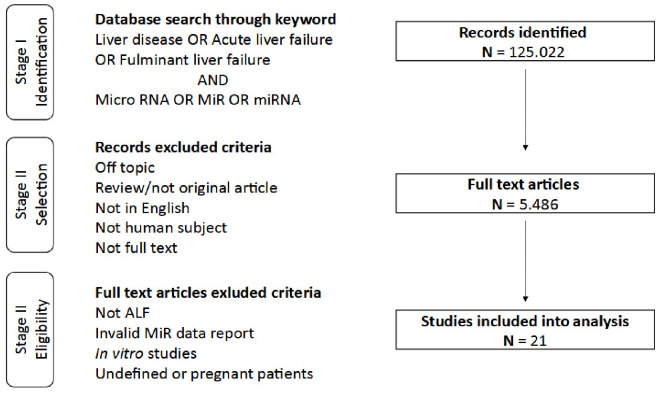
Flow chart of database searching and selection process.

The study concept was based on the assumption that the molecular signature of the ALF in the blood may include molecules originating from peripheral organs, including the liver and the brain. First, we performed a systematic search in the electronic database MEDLINE via Pubmed access [www.ncbi.nlm.nih.gov/pubmed, accessed on 22 November 2021] and Elsevier Scopus [www.scopus.com, accessed on 22 November 2021] for original publications published from 1 January 2011 up to 1 July 2021 focusing on ALF patients and miRNA expression studies using subjectively selected keywords based on literature revision and medical subject heading. The summary of searching steps underlying our systematic review is presented in Figure 1. Two authors of the review (K.O.G. and K.M.) independently extracted data using eligibility criteria and removed duplicates. To unify the data based on full-text screening, we excluded studies on patients with unclear ALF diagnosis (e.g., relation to chronic liver disease, multifactorial disease problems interfering with ALF, such as alcohol history, liver transplantation, or pregnancy (Figure 1)).

After identification of repeatedly reported differential blood-based miRNAs, in the next step, we revised reports of the differential miRNA profiles in the liver and urine with a search for the overlap between the miRNA alterations in these specimens. Thus, we verified the number of up and down-regulated miRNAs in serum and plasma samples and the number of miRNAs reported to be both up and down-regulated. Finally, we addressed the questions of ALF specificity and miRNAs functional implications in the pathogenesis of the disease. To this end, we performed a computational prediction of genes and pathways regulated by the selected miRNAs.

MiRNAs target genes and biological pathways analysis were performed using freeware tools available on DIANA website (http://diana.imis.athena-innovation.gr, accessed on 22 November 2021). Micro-T tool based on the miRBase database was used to identify potential miRNA target genes and pathways. TargetScan was used to study the prediction of the potential target genes and demonstrate relationships between them. The MiRPath v3.0 pathways analysis was performed to verify the involvement of selected genes in biological pathways according to the Kyoto Encyclopedia of Genes and Genomes (KEGG, http://www.genome.ad.jp/kegg, accessed on 22 November 2021), [47]. MiRPath was also used to visualize the clustering of the selected miRNAs based on their influence on molecular pathways. The Diana tool enables clustering miRNAs dependent on the subset which targets regulated pathways and the significance level of the interaction. Heatmaps used for visualization and other graphs were performed using licensed Prism 5 software by GraphPad, San Diego, CA, USA.

## 3. Research Summary

**Figure 2:**
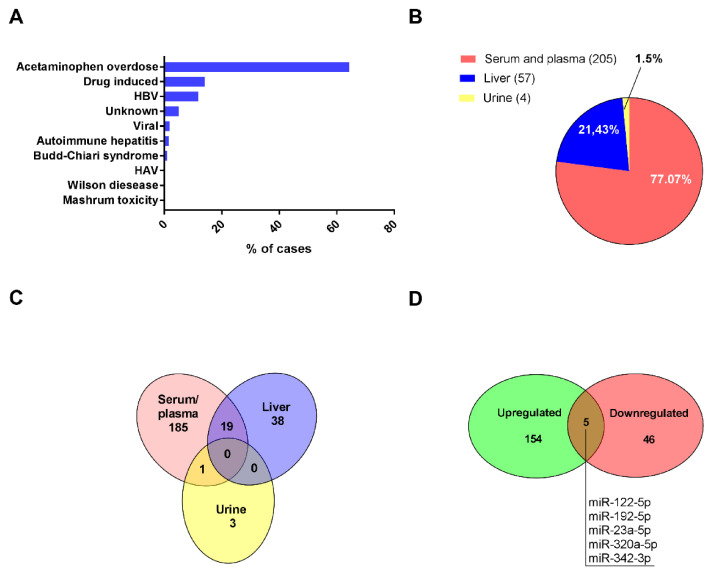
Characteristics of the last decade’s studies on differential miRNAs in ALF. Etiology of ALF (panel (**A**)). Pie chart presenting the number of differently expressed miRNAs in the referred specimens (panel (**B**)). Venn diagram showing overlapping of differently expressed miRNAs (panel (**C**)). The number of up and down-regulated miRNAs in serum and plasma samples. Overlapped miRNAs were regulated both up and down as was based on selected publications (panel (**D**)).

Database search resulted in 5486 full-text references. After removing duplicates, not-related, not-human, or the in vitro studies, articles were screened for eligibility (Figure 1). As a result, 21 publications covering data collected from a total number of 852 patients from seven different countries were included in our analysis (Table 2). As mentioned in the introduction, the etiology of the ALF is much varied. The causes of ALF are summarized in Figure 2 panel A.

The etiological distribution of ALF cases delivered from our study search corresponds well to clinical data described in the literature [68,69]. In total, 78% of patients suffered from drug-induced ALF, the vast majority were acetaminophen overdose cases (548 patients). Various viral infections were a direct cause of 14% of ALF, whereas only 7% of ALF causes were due to other conditions that included almost 5% of undetermined causes. The rarest single reports cases were Hepatitis A, Wilson disease, and mushroom *Amanita phalloides* poisoning.

MiRNAs expression data were mostly derived from blood samples (serum or plasma) of ALF patients. Five studies described miRNAs detected in ALF liver biopsies predominantly taken from HBV patients due to their significant diagnostic value in hepatitis management [70]. A single study evaluated miRNAs levels in the urine of ALF patients in relation to serum [65], (Table 2 and Figure 2, panel B).

In total, 246 differentially expressed miRNAs were reported in selected publications. The number of 217 miRNAs, were identified in a single study. Seven of the studies detected single miRNAs, while 4 of the studies used microarray or quantitative real-time polymerase chain reaction (qPCR) to recognize the large patch of miRNAs sequences (Table 2). Studies by Krauskopf et al., 2017 [54], 2020 [55] used the next-generation sequencing (NGS) technique to interrogate global miRNA signature. The advantage of NGS technology is its ability to detect the whole miRNome, including structural modifications of miRNAs, also called iso-miRNAs [54,55]. Due to the variety of techniques used in the referred studies, a quantitative single miRNA expression meta-analysis was not able to be performed. Data presenting all differently expressed miRNAs reported in patients with ALF, including information about the direction of observed miRNA level change (Up or Down), miRNA family membership, ALF cause, and respective reference, are shown in Supplementary Tables S1 and S2. It is noteworthy that the descriptions of some miRNAs used in the publications do not match the current nomenclature requirement due to the lack of a descriptor tag indicating from which double-stranded RNA the sequence comes from (3p/5p) or miRNA identity was uncertain. This fact, unfortunately, disallows the input of such data into the online database. These miRNAs are included in Supplementary Tables S1 and S2 and marked with a star.

The Venn diagram demonstrates the distribution of miRNAs in evaluated specimens (Figure 2, panel C). From 205 miRNAs differently expressed in ALF serum/plasma, only 19 overlap with 57 miRNAs detected in liver biopsies. In turn, from four miRNAs (miR-9-3p; miR-320a; miR-375, and miR-940) described in the urine of ALF patients, only miR 320a was also found upregulated in the serum. From miRNAs differently expressed in ALF patients serum/plasma 75% were upregulated since for five different miRNAs direction of change was inconsistent throw-out analyzed publications (Figure 2, panel D).

**Figure 3:**
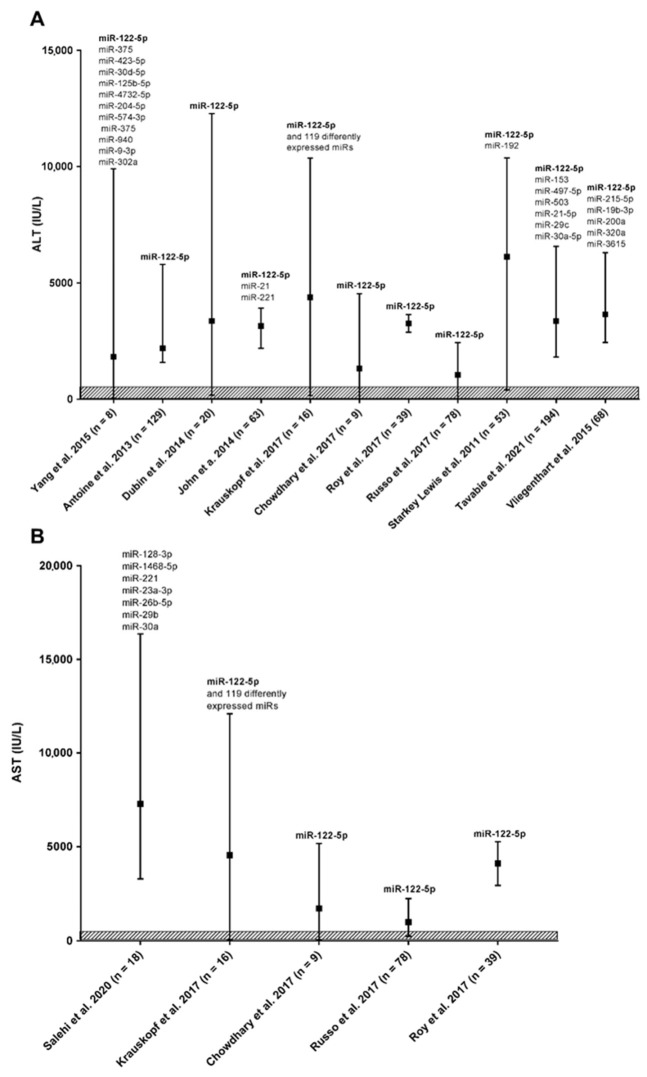
ALT (panel (**A**)) and AST (panel (**B**)) activity in serum of ALF patients with upregulated miRNAs. Black squares represent medians, bars range of measured ALT activity. Above the bars are listed differently expressed miRNAs reported in each cohort. The norm value for ALT/AST activity in blood for people without risk factors for liver disease in international units per liter (IU/L), according to the American College of Gastroenterology, is indicated as a patterned box.

Despite the evident progress in current methodologies, diagnosis of ALF still has several limitations and relies on verification of liver enzymes in the patients’ blood. As an effort to better integrate miRNAs data into a full clinical picture, we established relations between miRNAs (represented in Table 3) and serum biochemical parameters. In our study, serum biochemical data were available from 16 publications covering 740 patients with ALF. Three of the studies also confirmed HE using the WHC scale [58,59,61]. We analyzed the clinical parameters of ALF patients and correlated the two most consistently evaluated parameters: ALT and AST plasma levels, to differently expressed miRNAs observed in each study (Figure 3).

This comparison revealed wide dispersion in measured enzyme activity between patients inside the group and matched side to side cohorts. This is not surprising taking into account a divergent etiology, clinical appearance of ALF, and variable analytical methods and instruments used. More importantly, approximately 95% of evaluated ALF patients presented much higher ALT/AST activity in the blood than is considered as norm range by American College of Gastroenterology guidelines [71]. Regarding evaluated miRNAs, only miR-122-5p has been explored in most of the studies, and, importantly in all cases, upregulation of miR-122-5p accompanied the ALT/AST activity increase. This clearly indicates its potential as a biomarker in human ALF.

Understanding and analysis of miRNAs data gathered by various techniques and, without row data available, are quite problematic. In subsequent steps, we decided to exclude miRNAs that were reported ones or sequences not precisely defined. We focused on serum and plasma samples due to the majority of available data and the potent diagnostic role of circulating miRNAs. Thus, we selected a set of altered 25 miRNAs repetitively reported in ALF patients’ blood in a minimum of two independent reports (Table 3). Of selected 25 miRNAs, miR-122-5p was referred in 14 publications, whereas 15 miRNAs were reported in only two publications.

Across miRNAs assessed in this study, notable trends in the genomic spread of dysregulated miRNA genes were observed. Firstly, the dysregulated miRNAs were seen across multiple chromosomes. No defined chromosomes were showing a higher proportion of their miRNAs becoming dysregulated during ALF. Selected miRNAs were distributed on 17 different chromosomes including chromosome X (Table 3). Analysis of ALF-related miRNAs revealed seven miRNAs that have cluster organization (cluster members are shown in Table 3). Furthermore, miR-23b and miR-24-1 localized on chromosome 9 belong to the same cluster thus are probably expressed in an interdependent manner. We also checked Supplementary Table S1 for the occurrence of miRNA cluster members listed in Table 3. Indeed, four more cluster members were elevated in ALF patients: miR-103b, miR-301b, miR-194, and miR-27b suggesting the possibility of common upregulation of the whole miRNAs clusters.

**Figure 4:**
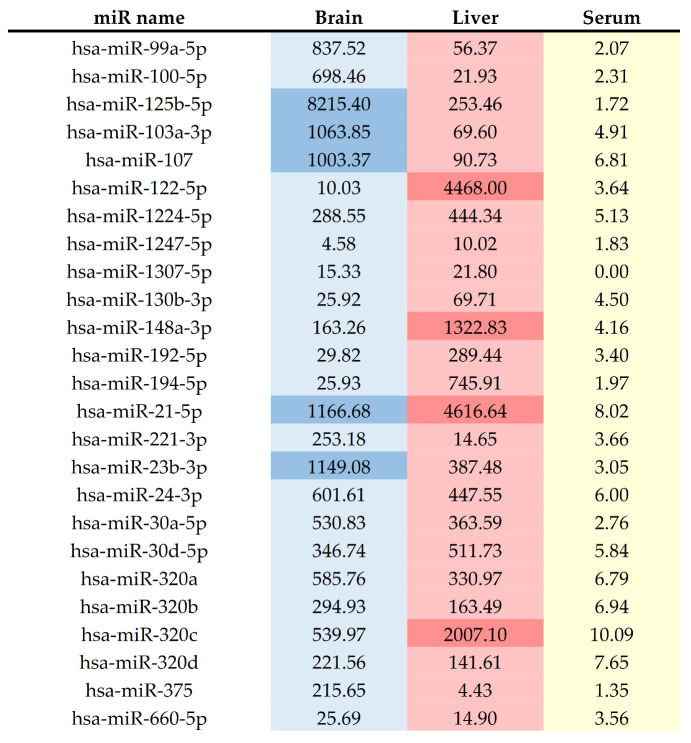
Normalized miRNA expression level in the normal human brain, liver, and serum analyzed by Human miRNATissueAtlas of miRNAs expression. MiRNAs with distinctive high expression are highlighted.

Considering that the spectrum of ALF symptoms includes both liver and brain alterations, we checked the tissues’ distribution pattern of selected miRNAs using Human miRNATissueAtlas [https://ccb-web.cs.uni-saarland.de/tissueatlas/patterns, accessed on 22 November 2021]. Among the selected 25 miRNAs elevating in the ALF patients’ blood, miR-122-5p, miR-21-5p, miR-320c, and miR-148 represented highly specific liver expression. In turn, miR-125b-5p, miR-103a-3p, miR-107, miR-99a-5p, and miR-100-5p were more abundant in human brain tissue compared to the liver (Figure 4). The normalized expression of ALF-induced miR-125b-5p was two-fold higher than the miR-122-5p expression level indicated in the liver, a well-proven example of a high tissue-specific miRNA sequence [73,74]. Other miRNAs presented relatively low and comparable expression patterns in both organs. We have also verified selected miRNAs regarding their plasma and serum expression levels. Worth noting here, in normal conditions, highly specific for the liver or the brain miRNA: miR-122-5p and miR-125b-5p are in the plasma at a relatively low level. The significant increase in miRNAs expression level observed in ALF patients might suggest tissue impairment. Of note, in this context, the role of circulating miR-122-5p as a marker of liver damage was previously widely discussed [73,75,76]. However, to our knowledge, there are no data documenting alterations of circulating miRNAome and CNS impairments observed as a result of liver failure.

Equalized enrichment of liver and brain-origin miRNAs in the blood samples of ALF patients, prompted us to formulate the hypothesis of the miRNAs-dependent axis between liver and brain. Moreover, ALF-caused alterations in CNS and/or impaired BBB integrity may induce the release of brain-specific miRNAs into the circulatory system reflecting CNS status and ALF molecular signature.

**Figure 5:**
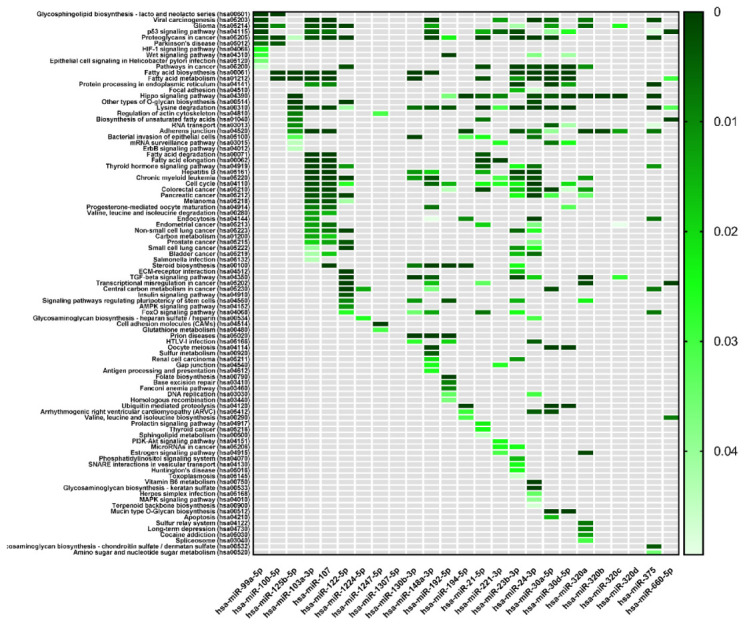
Heatmap of significant pathways predicted by DIANA-miRPath (v.3.0) for twenty-seven differentially expressed miRNAs in serum of ALF patients. KEGG pathways classification analysis of differentially expressed miRNAs is presented on the y-axis and miRNAs on the x-axis. The color intensity represents the p-value with dark green as the most significant predicted miRNA-pathway interactions (*p*  <  0.05 in Fisher’s Exact Test).

The most significant pathways prediction for twenty-five differentially expressed miRNAs in serum of ALF patients was performed and visualized as a single color scale heatmap (Figure 5). The biological pathways were predicted with mirPath v3.0., after setting up the target prediction threshold on a 0.8. As a result, 94 significant KEGG pathways were found (Figure 5).

KEGG pathways cover a large number of genes reflecting a diversity of biological processes. However, the expression of genes within particular pathways provides a way to estimate the activity of certain biological processes.

**Figure 6:**
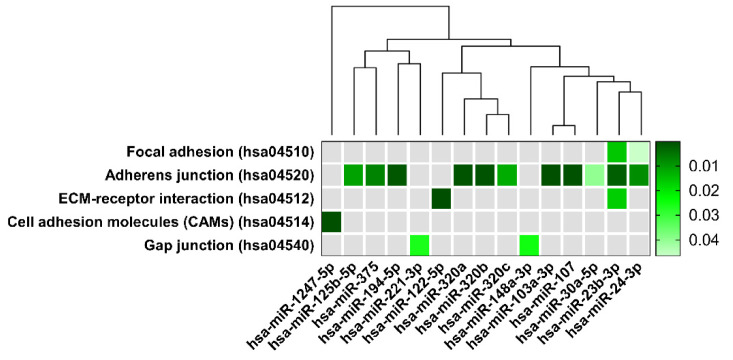
Heatmap of selected pathways predicted by DIANA-miRPath miRNA with hierarchical cluster dendrogram achieved by the linkage clustering method via TarBase. KEGG pathways classification analysis of differentially expressed miRNAs is presented on the y-axis and miRNAs on the x-axis. The color intensity represents the p-value with dark green as the most significant predicted miRNA-pathway interactions (*p*  <  0.05 in Fisher’s Exact Test).

Worth noting, five of the identified pathways associated with 15 of the analyzed miRNAs belongs to the same KEGG pathway functional group called “Cellular community” containing: Focal adhesion (hsa04520); Adherens junction (hsa04530); Tight junction (hsa04540); and Gap junction (hsa04550) (Figure 6).

We designed a dendrogram grouping miRNAs related to Cellular community pathways according to their biological function regarding miRNAs family membership (Figure 6). The branch lengths represent a difference between miRNAs and show variability in miRNAs’ abundances in their regulation of specific pathways. A dendrogram revealed strong relation between miR-103a-3p and miR-107 in controlling the adherens junction pathway. On the other hand, it displayed a distinct CAMs pathway regulating miR-1247 from the other evaluated miRNAs.

The only member of the cellular community pathways group not predicted in DIANA-miRPath analysis was a “Tight junction”. However, this pathway covers the inter-alia tight junction protein 1 (*TJP1*) gene, which is also a significant part of the Adherens junction pathway and a predicted target of miR-23b-3p and miR-320a (Table 4). Tight junction KEGG pathway contains also Cingulin/RhoA/Rock signaling path regulating Zonula occludens-1 barrier formation in endothelial tight junctions [77]. Genes of those three signaling proteins are potentially controlled by miRNAs included in our analysis (miR-125-3p for Cingulin; miR-122-5p, miR-375 for RhoA, and miR-148a for Rock) [78,79,80,81]. Moreover, a significant amount of scientific reports convincingly point out the role of miR-122-5p in the regulation of occludin expression, one of the most prominent tight junction proteins [82,83,84]. Those merely points to the need for a deeper discussion of the results obtained using a bioinformatics algorithm.

The link between gene pathways related to intercellular communication and ALF-induced miRNAs may indicate its involvement in the pathomechanism of ALF. Indeed, hepatic decomposition referred to as a loss of intercellular integrity, easily recognized during histological evaluation, is one of the marks of acetaminophen toxicity [85,86,87].

The “Cellular community” pathways encompass genes coding proteins strongly associated with the BBB structure and function [31]. Considering KEGG pathways obtained from our analysis, the adherens junction pathway (hsa:04514), is related to most selected miRNAs (Figure 6). In the context of BBB function and structure maintaining, the adherens junction pathway (hsa:045020), with cell adhesion molecules pathway (hsa:04514), including *CDH2* gene coding for cadherin 2 involved in the regulation of protein localization to adherens junction, is critically involved. Adherens junction is a cell–cell junction composed of the epithelial cadherin–catenin complex at which the cytoplasmic face of the plasma membrane is attached to actin filaments. The adherens junction pathway genes control vascular permeability [88] and brain vascular homeostasis [89]. In our study hsa:045020 pathway constitutes the majority (more than 40 defined genes; Table 4). Among them, genes: *ACTG1*, *ACTB*, *ACTB1*, and *ACTN1* encoding different isoforms of actin (β and γ actin) [90], are critical for actin cytoskeleton changes linked with the functioning of several cellular processes (cell shape maintenance, chemotaxis, cell movement, adhesion, and other). Other genes: *CTNNB1*, *CTNNA1*, *CTNND1* coding isoforms of catenin proteins, form a complex that constitute adherens junctions and are necessary for the maintenance of epithelial cell layers by regulating cell growth and adhesion. The catenin proteins also anchor the actin cytoskeleton. The adhesion molecules of nectin family genes: *PVRL4*, *PVRL1*, *PVRL3* are involved in the formation of cell–cell junctions, including adherens junctions and synapses. The cytoskeletal proteins vinculin and tight junction protein 1, coded by *VIN* and *TJP1*, respectively, regulate adherens junctions, are associated with cell–cell and cell–matrix junctions, and are involved in anchoring F-actin to the membrane. Tight junctions regulate the movement of ions and macromolecules between endothelial and epithelial cells. The large groups of genes are engaged in signaling processes that include genes coding different types of receptors of tyrosine phosphatase (*PTPRJ*, *PTPRB*, *PTPRF*, *PTPN1*), tyrosine kinase 2 (*ERBB2*), mitogen-activated protein kinases (*MAP3K7*, *MAPK3*, *MAPK1*), and genes associated with transforming growth factor-β signaling pathway (*SMAD2*, *SMAD3*, *SMAD4*, *TGFBR1*, *TGFBR2*), one of the most important and complex signaling pathways in vascular development and homeostasis of the brain vessel. The TGF-β signaling pathway dysfunction has been repeatedly linked to various cerebrovascular diseases [91,92,93] and implicated in BBB formation and permeability control by regulating tight and adherens junctions [94,95,96].

The loss of intercellular structural components is linked with the cytoplasmic side of focal adhesions molecules (hsa:04520) pathway comprising genes coding proteins forming large molecular complexes linking transmembrane receptors (e.g., integrins). One of a hallmark of focal adhesions is the clustering of integrins to the actin cytoskeleton and mediating intercellular signaling. These heterogeneous complexes are highly dynamic structures and are targeted by regulatory signals of proteins *GSK3B*, *PI3K*, *PTEN*, *JUN*, *RHOA*, *PAK2*, and *PAK3*. At the brain vasculature level, direct signal-transducing regulates cerebrovascular morphogenesis and endothelial barrier integrity. Recent reports have shown that focal adhesion kinase (*PTK2*) colocalizes with the tight junction proteins occludin and Zonula occludens-1 (*ZO-1*) [97]. The protein coded by the *PTK2* gene is also one of the key molecules that negatively regulate RhoA activity, an important regulator of BBB integrity [98]. In this context, studies have demonstrated that increased RhoA activity leads to disassembly of the complexes within cell-cell junctions and subsequent barrier disruption [96,97]. The proteins coded by *VEGFA*, *-B*, *-C* genes are members of the vascular endothelial growth factor family, best known as key regulators of angiogenesis. VEGFs play a role in blood and lymph vessel development and homeostasis. Particularly, *VEGFA* is involved in angiogenesis and vasculogenesis, and *VEGFC* mediates mostly lymphangiogenesis but also has angiogenic activity [99]. No less importantly, the gene coding for vasodilator-stimulated phosphoprotein (*VASP*) is associated with filamentous actin formation, and likely plays a widespread role in cell adhesion and motility. *VASP*, which function is regulated by the cyclic nucleotide-dependent kinases PKA and PKG may also be involved in the intracellular signaling pathways that regulate integrin–ECM interactions [100].

Consequently, the extracellular matrix (ECM)-receptor interaction (hsa:04512) pathway was highlighted in our analysis (Figure 6). The pathway is relevant for neural tissue, in particular by composing a cellular architecture, synapse arrangement, and stabilization. In addition, the hsa:04512 pathway may be of importance in the regulation of neuroplasticity and memory formation [101]. In total, 18 different reported genes were annotated in our study into this pathway (Table 4). They include 3 member genes of integrin heterodimeric transmembrane receptors composed of alpha and a beta subunit: *ITGB1*, *ITGB8*, *ITGA6*, all of which are involved in cell adhesion and signaling. Next, collagenous (6 members of collagen family: *COL4A1*, *COL4A2*, *COL5A1*, *COL4A3*, *COL6A1*, *COL27A1*) and non-collagenous, the extracellular matrix glycoproteins (5 members of laminin family: *LAMC1*, *LAMC2*, *LAMC3*, *LAMB1*, *LAMA5*), constituents of the basement membranes with TNXB gene coding tenascin-X controlling production and assembly of collagen. Collagen isoforms *COL4A1* and *COL4A2*, defined in a GWAS analysis of cerebral small vessel disease as mainly expressed in the brain endothelial cells [93]. Collagens and laminins, implicated in a wide variety of biological processes, regulate cell adhesion, signaling, neurite outgrowth, differentiation, migration, and metastasis. The abnormal expression of laminin family genes is associated with clinical outcomes of, for example, hepatocellular carcinoma [102].

Since, the ECM features are high concentrations of proteoglycans and glycosaminoglycans, of particular relevance are changes of heparan sulfate proteoglycans (HSPGs), a family of glycoproteins with a small heparan sulfate side chain. HSPGs, attached directly to the ECM, are secreted into the extracellular space, or on secreted vesicles [103]. HSPGs that are present throughout the neural ECM are potentially secreted by activated glial cells and seem to be associated with neurodegenerative diseases [104]. Interestingly, HSPGs are likewise involved in BBB organization [105] and control monocytes’ crossing the BBB. Agrin, encoded by the gene *AGRN*, is a large HSPG essential for neuromuscular synapse formation [106]. Finally, the *CD44* gene, coding for multifunctional cell surface adhesion receptor for hyaluronan or hyaluronic acid and several other ligands, including osteopontin, collagens, and matrix metalloproteinases [107], was indicated in our analysis. In line with the above, hyaluronan-mediated motility receptor (*HMMR* gene) was likewise defined. Another matricellular protein with multiple effects due to interacting with structural components of the ECM is thrombospondin 1 coded by the *THBS1* gene [108].

Within the Gap junction pathway (hsa04550), including the intercellular membrane channels facilitating the direct cytoplasmic exchange of small molecules between adjacent cells, apart from the *GJA1* gene encoding one of the most important connexin proteins, connexin-43 (Cx43), most of the genes constitute a family of genes encoding tubulins: *TUBB*, *TUBB6*, *TUBA8*, *TUBB2B*, *TUBB4B*. Tubulins compose microtubules, key cytoskeletal fibers, which regulate the shape, polarity, migration, and survival of the endothelial cells [109]. Tubulins posttranslational modifications, including acetylation, play an important role in the control of microtubule structure and microtubule-based cellular functions [110]. The family of genes encoding different subunits of G protein-coupled receptors (*GPRC*, *GNAS*, *GNA11*, *GNAI2*) is the largest family of cell surface receptors involved in signal transduction. The GPRC proteins, characterized by a 7-transmembrane domain structure with an extracellular N-terminus and an intracellular C-terminus, play roles in a variety of physiological processes, including immune responses, neurotransmission, cardiac function, and sensory functions and their aberrant activity or expression contributes to some of the most prevalent human diseases [111]. The GPRC proteins via RhoA activation induce cytoskeletal changes, important for cell migration. Another gene in the hsa04550 pathway, *GRB2*, coding for Growth Factor Receptor Bound Protein 2, is associated with Hepatitis C and Hepatitis E. In turn, Calmodulin-activated adenylate cyclases (*ADCY1* and *ADCY8* genes) generate cAMP. Collectively, despite indicated genes pathways that are important for BBB maintenance, a causational relationship between differential miRNA expression identified in body fluids and these features of BBB alterations needs to be established in future studies.

The current knowledge, based mostly on clinical imaging (MRI) and experimental studies, indicates persistent BBB dysfunction with increased paracellular barrier permeability in ALF patients [112]. The previous postmortem studies on the human brain revealed signs of both the cytotoxic and vasogenic components of ALF-induced edema [32]. BBB increased permeability in the subacute and chronic HE stages is significantly less visible than changes observed in acute HE when there can be uncontrolled extravasation of plasma proteins, leukocytes, and vasogenic edema formation [113]. Additionally, the signs of systemic or neuroinflammation during ALF or as a result of associated bacterial infection can disturb the integrity of the BBB. In line with a few studies referring to BBB alterations in ALF patients, more pieces of evidence were supplied from studies of animal models which documented BBB permeability changes upon ALF [113,114,115]. How BBB alterations and recovery occur in ALF is unclear. One hypothesized scenario suggests fast sealing of the barrier due to compensatory overexpression of BBB-composing proteins, the presence of which can be found along blood vessels in ALF-injured tissue. In addition to protein expression changes, BBB functioning is controlled by protein-protein interactions and signaling. Since the recovery of the BBB is a complex process, the final effect is dependent on the expression pattern of functional proteins, and involves the *de novo* organization of junction proteins. BBB components disorganization leads to increased para-cellular permeability. Whose duration, and intensity may override any effect described as increased transcellular permeability [114].

## 4. Concluding Remarks

The increase in the concentrations of liver metabolites reaching the CNS dominates in ALF pathology thus ALF alters the homeostasis of the liver-brain axis by a mechanism encompassing the expression changes of hundreds of genes responsible, i.e., for the antioxidant system, neurotransmitter biogenesis, inflammatory processes, to list a few. MiRNAs acting as modulators of gene expression seems to play a crucial but still not well-established role in this control. Therefore, a more detailed analysis of selected miRNAs in the context of signaling mediators and/or biomarker candidates for ALF offering new safety biomarkers that outperform current markers in terms of their sensitivity, specificity, and clinical predictivity is highly desirable.

The systematic analysis of 21 reports from the last decade, involving a total of 852 participants, determined 205 circulating miRNAs to be altered in the blood of ALF patients regardless of the study design and methodology. From this set, 25 miRNAs were repetitively altered in independent reports. We selected 25 miRNAs, emerging predominantly from the analyses of blood samples obtained from the acetaminophen-overdose group of patients, that represented the most promising biomarker candidates for a diagnostic panel for symptomatic ALF. A new miRNAs identification is the most promising alternatives for classic liver tests. In addition, the small RNAs are also attractive therapeutic targets due to their size, and relative availability of chemically designed active substances, in a safe manner used in targeting and inhibiting miRNAs.

Since acetaminophen-induced liver injury results in oncotic necrosis, hepatocyte-specific miRNAs elevate in the plasma within hours, such as miR-122-5p or miR-192-5p, proposed so far as a potential biomarker tool. The diagnostic potential of miR-122-5p to predict the subsequent onset of early liver injury was documented experimentally before the increase in ALT [115,116]. In preclinical studies, both miR-122-5p and miR-192-5p were enriched in liver tissue and presented dose- and exposure-dependent changes in plasma, paralleled with serum ALT/AST levels and the liver histology [115,116]. Although our systematic search revealed considerable coincidence of miR-122-5p elevation and ALT/AST increase in plasma of ALF patients throughout the studies, no other miRNAs seem to serve as a diagnostic tool.

The results of our analysis offer a selected group of miRNAs to assess whether pathognomonic “signatures” of circulating miRs could serve as a diagnostic tool. The defined common pathways targeted by selected miRNAs are highly relevant to cellular community processes and suggest that loss of intercellular structural and functional components may result in brain dysfunction with BBB impairment. However, a causational relationship between differential miRNAs expression identified in the circulation and clinical features observed in ALF patients has to be demonstrated in further study.

The difference between the criteria of scientific research and clinical application is quite obvious. Therefore, imperfect verification of potential miRNA biomarkers candidates may limit the study impact. An applicable biomarker must not only be significantly differentially expressed, but also be capable of defining the correlation with the outcome of patients. It is important to underline that the analyzed studies were differently designed and several cohort comparisons were made in each study. In general, the limited sample size or not an equal group of patients was the limitation of this study, thus a larger sample size cohort is needed for further assessment implementation. Moreover, miRNA detection could also be affected by measurement principle, method, instrument, and the researcher operation. Thus the sample size may be a critical point to guarantee the accuracy for detection. In addition, the absolute quantitative detection protocol may be crucial, as well as a correction of the circulating biomarker to healthy volunteers. In clinical application, the level of circulating biomarkers is under the influence of multiple individual classifications, including age, gender, lifestyle, and so on. Unfortunately, only very few reports performed validation experiments and used greater cohorts, which might be the reason for the limited reproducibility of the reported data.

In terms of the conceptual design, around 61.9% of the studies employed a candidate approach, 23.8% used high throughput technologies, and 9.5% combined these two strategies. miRNA profiles were determined usually by RT-qPCR. However, other methodologies, including NGS, or microarray were also used. Importantly, the methods used differ in terms of sensibility and accuracy, especially at single-base resolution.

For RNA extracted from biofluids, the source of microRNAs and the extraction method that may influence recovery and the outcome is substantial. RNA isolation kits were preferentially used over the manual methods. The normalization of the signal is another limitation since a normalizer choice is critical and challenging. In analyzed studies, the internal normalization controls for relative quantification in RT-qPCR were not identical. The universal endogenous control is unlikely to be discovered and a suitable reference should be assessed every time considering the different biological conditions of the samples.

The complete understanding of both positive and negative roles of miRNAs in the pathomechanism of ALF might be a problem. Highly specific to the brain tissue miRNAs may impair cell function, exacerbate brain injury-causing oxidative stress, neurotransmission, metabolism, neuroinflammation, attendant cell morphology, and cell composition, contributing to increased brain dysfunction. The literature revealed that, for example, increased miR-125b expression has been observed in the cerebellum, hippocampus, medial frontal gyrus [117], temporal lobe neocortex [118,119], and frontal cortex [120] in AD patients. Thus, observed in ALF upregulation of brain-specific miRNAs in the serum might reflect neuronal status. Additionally, released miRNAs may dependently or independently on the disease status affect microcirculation, endothelial activation, and lead to the BBB disturbance. Alternatively, after the BBB cross, miRNAs may initiate a cascade of neurological processes alterations mediated by oxidative stress, brain signaling, neuroinflammation, etc., to list a major. These concepts open an exciting new chapter on disease mechanisms and strategies for developing non-invasive tools to monitor the proneness and progression of the disease. It is noteworthy that miRNA identification may in the future contribute to elucidating the ALF pathogenesis via the liver-brain axis since the miRNAs signature of ALF clinical features (e.g., edema) is still missing. Among the newly available techniques, circulating miRNAs have an irrefutable potential for new biomarkers and expand our understanding of ALF pathophysiology. It is crucial in the future to elucidate the mechanisms governing the biogenesis, sorting, release, and uptake of secreted miRNAs. Other questions remaining to be addressed in the future are whether miRNAs convey physiologically important information for specific cells and whether secretion is a selective process. Determination of sufficient amounts of secreted miRNAs required for cell-to-cell signaling is essential to understanding the role of secreted miRNAs in the pathological processes regulation. Next, the secretion and incorporation of miRNAs are generally conserved phenomena that remain unexplored. Nevertheless, the miRNA-mediated form of inter-organ communication presents an open field for the understanding of signal and molecule transfer between cells. The elucidation of this information transfer system will be important in understanding many biological processes.

## Figures and Tables

**Table 1:** Etiology of ALF.

Cause	Causative Factor	Therapy	Frequencyin Adults *
Intoxication	Acetaminophen	N-acetyl cysteine	58%
	Isoniazid	Hydration	
	Other drug-driven liver damage		
	Mushrooms poisoning	Penicillin	
Viral	Hepatitis B/C/A/E/ **	Antiviral therapy	10%
Metabolic	Wilson disease	Copper chelation	7%
	Autoimmune hepatitis	Steroids	
	Acute fatty liver of pregnancy	Delivery of the fetus	
Other	Budd-Chiari syndrome	Surgery	5%
	Ischemic/sepsis shock	Hemodynamic stabilization	
	Heat stroke	Hydration	
Unknown	Undetermined etiology		14%

* According to Rajaram et al., 2018; ** Ordered by frequency of occurrence.

**Table 2:** Summary of the included studies.

Ref.	Year	Disease	Specimen	Region	N **	Analysis Method	Changed miRNAs	Notes
[48]	2013	Acetaminophen overdose	Plasma	UK	129	RT-qPCR	1	
[49]	2017	ALF *	Plasma/liver	USA	9/4	RT-qPCR	1	
[50]	2015	HBV	Liver	Italy	4/10	RT-qPCR	17	
[51]	2014	ALF *	Serum	USA	35/12	RT-qPCR	1	
[52]	2014	ALF *	Serum	Germany	63/15	RT-qPCR	3	
[53]	2015	Acetaminophen overdose	Serum	Netherlands	6/6	RT-qPCR, HiSeq 2000	3	
[54]	2017	Acetaminophen/HBV	Serum	USA	16/22	Illumina HiSeq 2000	132	
[55]	2020	Acetaminophen overdose	Serum	Netherlands	1	RT-qPCR, NGS	57	Case Study
[56]	2019	HBV	Liver	China	4/10	Microarray	38	
[57]	2017	ALF *	Serum/liver	USA	39/5	RT-qPCR	1	
[38]	2017	Drug induced ALF	Serum	USA	78/40	GeneChip^®^ 3.0 miRNA microarrays	1	
[58]	2020	Acetaminophen overdose	Serum	UK	18/undefined	RT-qPCR	7	HE grade diagnosed
[59]	2011	Acetaminophen overdose	Serum	UK	53/25	RT-qPCR	2	Controlled Clinical Trial/HE grade
[60]	2018	HBV	Liver	India	30/6	Microarray	1	
[61]	2021	Acetaminophen overdose	Serum	USA	194	RT-qPCR	7	HE grade diagnosed
[62]	2014	Acetaminophen overdose	Serum/plasma	USA	42/12	RT-qPCR	3	
[63]	2018	HBV	Serum	China	55	RT-qPCR	3	
[64]	2015	Acetaminophen overdose	Serum	UK	68	RT-qPCR (miScript System)	6	
[65]	2015	Acetaminophen overdose	Serum/urine	USA	8/10	Whole genome PCR array	12	
[66]	2016	Acetaminophen overdose	Serum/liver	Germany	9/4	RT-qPCR	1	same cohort as Chowdhary et al., 2017 [49]
[67]	2018	Acetaminophen overdose	Serum	USA	8/10	RT-qPCR	4	same cohort as Yang X et al., 2015 [65]

* Cases characteristics Chowdhary et al. [49], non-acetaminophen ALF (8 unknown, 1 mushroom); Dubin et al. [51], 20 acetaminophen, 6 autoimmune hepatitis, 4 drug-induced, 2 hepatitis A virus, 1 hepatitis B virus, 1 Epstein Barr virus; John et al. [52], viral hepatitis (NSR, n 5 4; SR, n 5 5), toxic liver injury (NSR, n 5 11; SR, n 5 12), Budd–Chiari syndrome (NSR, n 5 5; SR, n 5 0), Wilson’s disease (NSR, n 5 1; SR, n 5 0), autoimmune hepatitis (NSR, n 5 2; SR, n 5 2), and indeterminate etiology (NSR, n 5 19; SR, n 5 2); Roy et al. [57], 15 drug-induced toxicity, 3 Budd–Chiari Syndrome, 7 viral hepatitis, 3 autoimmune hepatitis, 11 unknown, and 10 livers biopsies from ALF patients. ** Number of patients/ number of healthy controls.

**Table 3:** Characteristics of miRNAs reported in at least two independent publications carried on ALF patients.

Family	MiRNA	Localization	Cluster Members	References
miR-10	miR-99a-5p	chr21		[54,55]
miR-10	miR-100-5p	chr11		[54,55]
miR-10	miR-125b-5p	chr11		[54,55,65,72]
miR-103	miR-103a-3p	chr20	miR-103b-2	[54,55]
miR-103	miR-107	chr10		[54,55]
miR0122	miR-122-5p	chr18	miR-122b	[49,53,54,65]
miR-1224	miR-1224-5p	chr3		[38,48,51,52,59,61,62,63,64]
miR-1247	miR-1247-5p	chr14		[54,55]
miR-1307	miR-1307-5p	chr10		[54,55]
miR-130	miR-130b-3p	chr22	miR-301b	[54,55]
miR-148	miR-148a-3p	chr7		[54,55]
miRR-192	miR-192-5p	chr11	miR-194-2; miR-6750; miR-6749	[54,55,64]
miR-194	miR-194-5p	chr1	miR-215	[54,55]
miR-21	miR-21-5p	chr17		[54,61,62]
miR-221	miR-221-3p	chrX		[52,54]
miR-23	miR-23b-3p	chr9	miR-27b; miR-3074; miR-24-1	[54,55]
miR-24	miR-24-3p	chr9	miR-27b; miR-3074; miR-23b	[54,55]
miR-30	miR-30a-5p	chr6		[54,61]
miR-30	miR-30d-5p	chr8		[55,65]
miR-320	miR-320a	chr8		[54,64,67]
miR-320	miR-320b	chr1		[54,67]
miR-320	miR-320c	chr18		[54,67]
miR-320	miR-320d	chr13		[54,67]
miR-375	miR-375	chr2		[55,65]
miR-188	miR-660-5p	chrX		[54,55]

**Table 4:** ALF-related miRNAs comprising cellular community KEGG pathways and its predicted gene targets.

KEGG Pathways	miRNA Name	Gene Name	*p*-Value
Focal adhesion	miR-125b-2-3p	*BRAF*, *GSK3B*, *ITGB1*, *LAMB1*, *THBS1*, *PAK2*, *VCL*, *RHOA*, *PAK3*, *FYN*, *PPP1R12A*, *JUN*, *DIAPH1*, *VEGFC*, *PTEN*, *BIRC2*	4.7 × 10^−2^
	miR-23b-3p	*TLN2*, *PRKCA*, *ACTN2*, *MYLK4*, *MET*, *ITGB1*, *FLNC*, *CRKL*, *THBS1*, *MYL12B*, *PAK2*, *TNXB*, *PP1CC*, *BCL2*, *EGFR*, *FYN*, *COL6A1*, *AKT2*, *ARHGAP35*, *PPP1R12A*, *MAPK9*, *PIK3R3*, *CCND1*, *CTNNB1*, *COL4A2*, *PAK6*, *PRKCB*, *LAMC1*, *PDK‘*, *LAMC2*, *ITGA6*, *VEGFA*, *PTEN*, *MAPK1*, *KDR*, *MYL12A*, *XIAP*, *COL4A1*, *PPP1CB*	1.5 × 10^−2^
	miR-24-3p	*ACTB*, *GSK3B*, *ITGB1*, *ITGB8*, *FLNC*, *LAMA5*, *PIK3CB*, *CAV1*, *PXN*, *RAF1*, *BCAR1*, *EGFR*, *MYLK2*, *CAV2*, *ITGB5*, *ARHGAP35*, *PTK2*, *ITGA11*, *JUN*, *PIK3R3*, *CTNNB1*, *DIAPH1*, *FLNB*, *FLT1*, *FLNA*, *RAC1*, *LAMC1*, *VASP*, *FN1*, *TNC*, *BIRC3*, *PDPK1*, *VEGFA*, *TLN1*, *KDR*, *LAMA4*	4.6 × 10^−2^
Adherens junction	miR-125b-5p	*ERBB2*, *ACTG1*, *IQGAP1*, *MLLT4*, *CTNNB1*, *CTNNA1*, *WASF2*, *INSR*, *FGFR1*	10^−2^
	miR-103a-3p	*ACTB*, *CTNND1*, *ACTG1*, *IQGAP1*, *IGF1R*, *MLLT4*, *SMAD4*, *CTNNB1*, *CTNNA1*, *ACTN1*, *WASF2*, *CSNK2A1*, *FARP2*, *PTPRJ*, *CDC42*, *PTPRB*, *MAP3K7*, *CREBBP*, *TGFBR2*, *PVRL1*	1.2 × 10^−3^
	miR-107	*ACTB1*, *CTNND1*, *ACTG1*, *IQGAP1*, *IGF1R*, *PTPRF*, *MLLT4*, *DMAD4*, *CTNNB1*, *CTNNA1*, *ACTN1*, *WASF2*, *CSNK2A1*, *FARP2*, *PTPRJ*, *MAPK3*, *CDC42*, *PVRL3*, *PTPRB*, *MAP3K7*, *CREBBP*, *TGFBR2*, *PVRL1*	3 × 10^−4^
	miR-194-5p	*TGFBR1*, *WASL*, *ACTG1*, *LMO7*, *IGF1R*, *PVRL4*, *RAC1*	9.6 × 10^−4^
	miR-23b-3p	*ACTN2*, *CSNK2A2*, *MET*, *CTNND1*, *LMO7*, *IQGAP1*, *SMAD3*, *EGFR*, *TJP1*, *FYN*, *SMAD4*, *CTNNB1*, *WASF2*, *CSNK2A1*, *FARP2*, *PTPRJ*, *YES1*, *MAPK1*, *TGFBR2*	1.9 × 10^−3^
	miR-24-3p	*ACTB*, *CSNK2A2*, *SMAD2*, *PVRL2*, *SMAD3*, *EGFR*, *PTPRF*, *NLK*, *CTNNB1*, *CTNNA1*, *WASF2*, *RAC1*, *EP300*, *YES1*, *CREBBP*, *TGFBR2*, *PVRL1*	8.4 × 10^−3^
	miR-30a-5p	*CSNK2A2*, *MET*, *WASL*, *CTNND1*, *SMAD2*, *PTPN1*, *IGFR1*, *EGFR*, *NLK*, *RAC1*, *PVRL3*, *MAPK1*, *MAP3K7*, *TGFBR2*	3.9 × 10^−2^
	miR-320a	*SMAD2*, *SMAD3*, *IGF1R*, *VCL*, *TJP1*, *CDH1*, *CTNNB1*, *RAC1*, *INSR*, *SSX2IP*, *MAPK1*, *TGFBR2*	5.2 × 10^−4^
	miR-320b	*SMAD3*, *TJP1*, *CDH1*, *CTNNB1*, *RAC1*, *INSR*, *SSX2IP*, *MAPK1*	4 × 10^−4^
	miR-320c	*SMAD3*, *TJP1*, *CDH1*, *CTNNB1*, *INSR*, *SSX2IP*, *MAPK1*	1.2 × 10^−3^
	miR-375	*ERBB2*, *IQGAP1*, *PTPN1*, *IGF1R*, *PTPRF*, *RHOA*, *CTNNA1*, *WASF2*, *CDC42*, *PARD3*	6.9 × 10^−4^
ECM-receptor interaction	miR-122-5p	*ITGB1*, *ITGB8*, *LAMB1*, *LAMA5*, *COL27A1*, *AGRN*, *LAMC3*, *COL4A2*, *COL5A1*, *COL4A3*, *CD44*	8.3 × 10^−8^
	miR-23b-3p	*ITGB1*, *THBS1*, *TNXB*, *COL6A1*, *COL4A2*, *LAMC1*, *LAMC2*, *ITGA6*, *HMMR*, *COL4A1*	1.6 × 10^−2^
Cell adhesion molecules	miR-1247-5p	*CDH2*	9.6 × 10^−7^
Gap junction	miR-148a-3p	*ADCY1*, *ADCY8*, *GNAS*, *MAP2K5*, *RAF1*, *TUBB*, *TUBB6*, *CDK1*, *KRAS*, *TUBA8*, *GNA11*, *TUBB2B*, *SOS1*, *GNAI2*, *HTR2C*, *TUBB4B*, *MAPK1*, *GRB2*, *GNAI1*	2.4 × 10^−2^
	miR-221-3p	*RAF1*, *CDK1*, *PDGFD*, *GNAI2*, *GJA1*	2.6 × 10^−2^

## Data Availability

Not applicable.

## Supplementary Materials

The following are available online at https://www.mdpi.com/article/10.3390/ijms23010224/s1.
