# Supplementary material for: Insight into microRNAs-Mediated Communication between Liver and Brain: A Possible Approach for Understanding Acute Liver Failure?
Source: Int J Mol Sci. 2021 Dec 25;23(1):224. doi: 10.3390/ijms23010224 (PMC8745738; doi:10.3390/ijms23010224)
Supplement: Supplementary file 1 [file ijms-23-00224-s001.zip › Table S1.pdf]

Table S1. List of differently expressed MiRs in ALF patients.

MiR family	MiR	Direction of change	Patients characteristic	Reference
**miR-10/100**	miR-10a-5p	Up	Acetaminophen	Krauskopf et al. 2020
	miR-10b-5p	Up	Acetaminophen	Krauskopf et al. 2020
	miR-99a-3p	Up	Acetaminophen	Krauskopf et al. 2017
		Up	HBV	Krauskopf et al. 2017
	99a-5p	Up	Acetaminophen	Krauskopf et al. 2017
		Up	HBV	Krauskopf et al. 2017
		Up	Acetaminophen	Krauskopf et al. 2020
	miR-99b-5p	Up	Acetaminophen	Krauskopf et al. 2020
	miR-100-5p	Up	Acetaminophen	Krauskopf et al. 2017
		Up	HBV	Krauskopf et al. 2017
		Up	Acetaminophen	Krauskopf et al. 2020
	miR-125a-5p	Up	Acetaminophen	Krauskopf et al. 2020
	miR-125b-1-3p	Up	Acetaminophen	Krauskopf et al. 2020
		Up	Acetaminophen	Krauskopf et al. 2017
	miR-125b-2-3p	Up	Acetaminophen	Krauskopf et al. 2017
	miR-125b-2-3p	Up	HBV	Krauskopf et al. 2017
	miR-125b-5p	Up	Acetaminophen	Krauskopf et al. 2017
		Up	Acetaminophen	Yang X et al. 2015
		Up	Acetaminophen	Yang D et al. 2016
		Up	Acetaminophen	Krauskopf et al. 2020
		Up	HBV	Krauskopf et al. 2017
**miR-101**	miR-101-3p	Down	Acetaminophen	Krauskopf et al. 2020
**miR-103/107**	miR-103a-3p	Up	Acetaminophen	Krauskopf et al. 2020
		Up	Acetaminophen	Krauskopf et al. 2017
	103b	Up	Acetaminophen	Krauskopf et al. 2020
	miR-107	Up	Acetaminophen	Krauskopf et al. 2020
		Up	Acetaminophen	Krauskopf et al. 2017
**miR-122**	miR-122-3p	Up	HBV	Krauskopf et al. 2017
	miR-122-5p	Up	Acetaminophen	Krauskopf et al. 2017
		Up	ALF**	Chowdhary et al. 2017
		Up	HBV	Krauskopf et al. 2017
		Up	Acetaminophen	Yang X et al. 2015
		Up	Acetaminophen	Krauskopf et al. 2015
		Up	Acetaminophen	Antoine et al. 2013
		Up	Acetaminophen/HCV	Dubin et al. 2014
		Up	ALF**	John et a. 2014
		Up	Acetaminophen	Tavabie et al. 2021
		Up	ALF**	Russo et al. 2017
		Up	Acetaminophen	Starkey Lewis et al. 2011
		Down	Acetaminophen	Vliegenthart et al. 2015
		Up	HBV	Wen et al. 2018
		Up	Acetaminophen	Ward et al. 2014
	miR-1224-5p	Up	Acetaminophen	Krauskopf et al. 2020
	miR-1224	Up	ALF**	Roy et al. 2017

	miR-1224	Up	Acetaminophen	Krauskopf et al. 2017
	miR-1247-5p	Up	Acetaminophen	Krauskopf et al. 2020
		Up	Acetaminophen	Krauskopf et al. 2017
	miR-126*	Down	Acetaminophen	Krauskopf et al. 2017
**miR-128**	miR-128-3p	Up	Acetaminophen	Salehi et al. 2020
	miR-1249-3p	Down	Acetaminophen	Krauskopf et al. 2017
	miR-1260a	Down	Acetaminophen	Krauskopf et al. 2017
	miR-1260b	Down	Acetaminophen	Krauskopf et al. 2017
	miR-1285-5p	Up	Acetaminophen	Krauskopf et al. 2017
	miR-1290	Up	Acetaminophen	Krauskopf et al. 2017
	miR-1291	Up	Acetaminophen	Krauskopf et al. 2017
	miR-1303	Up	Acetaminophen	Krauskopf et al. 2017
	miR-1304-3p	Down	Acetaminophen	Krauskopf et al. 2017
	miR-1306-5p	Down	Acetaminophen	Krauskopf et al. 2017
	miR-1307-5p	Up	Acetaminophen	Krauskopf et al. 2017
		Up	Acetaminophen	Krauskopf et al. 2020
**miR-130**	miR-130a-3p	Up	Acetaminophen	Krauskopf et al. 2020
	miR-130a-5p	Up	Acetaminophen	Krauskopf et al. 2017
	miR-130b-3p	Up	Acetaminophen	Krauskopf et al. 2020
		Up	Acetaminophen	Krauskopf et al. 2017
	miR-130b-5p	Down	Acetaminophen	Krauskopf et al. 2017
	miR-140-3p	Up	Acetaminophen	Krauskopf et al. 2020
	miR-1468-5p	Up	Acetaminophen	Salehi et al. 2020
**miR-146**	miR-146a-5p	Up	Acetaminophen	Krauskopf et al. 2020
	miR-146b-5p	Up	HBV	Wen et al. 2018
**miR-148/152**	miR-148a-3p	Up	Acetaminophen	Krauskopf et al. 2017
		Up	Acetaminophen	Krauskopf et al. 2020
	miR-148a-5p	Up	HBV	Krauskopf et al. 2017
	miR-150-5p	Down	Acetaminophen	Krauskopf et al. 2017
	miR-152-3p	Up	Acetaminophen	Krauskopf et al. 2017
**miR-153**	miR-153*	Up	Acetaminophen	Tavabie et al. 2021
**miR-15/16**	miR-497-5p	Up	Acetaminophen	Tavabie et al. 2021
	miR-503*	Up	Acetaminophen	Tavabie et al. 2021
	miR-190a-5p	Down	HBV	Krauskopf et al. 2017
	miR-191-3p	Down	Acetaminophen	Krauskopf et al. 2017
	miR-191-5p	Down	Acetaminophen	Krauskopf et al. 2017
		Down	Acetaminophen	Krauskopf et al. 2020
**miR- 192/215**	miR-192*	Up	Acetaminophen	Starkey Lewis et al. 2011
	miR-192-5p	Up	Acetaminophen	Krauskopf et al. 2017
		Up	HBV	Krauskopf et al. 2017
		Up	Acetaminophen	Krauskopf et al. 2020
		Down	Acetaminophen	Vliegenthart et al. 2015
	miR-193*	Up	Acetaminophen	Krauskopf et al. 2020
**miR-193**	miR-193a-3p	Up	Acetaminophen	Krauskopf et al. 2017
	miR-193b-3p	Up	Acetaminophen	Krauskopf et al. 2017
	miR-193b-5p	Up	HBV	Krauskopf et al. 2017

	miR-193b-5p	Up	Acetaminophen	Krauskopf et al. 2017
	miR-194-3p	Up	HBV	Krauskopf et al. 2017
**miR-194**	miR-194-5p	Up	Acetaminophen	Krauskopf et al. 2017
	miR-194-5p	Up	HBV	Krauskopf et al. 2017
		Up	Acetaminophen	Krauskopf et al. 2020
	miR-19a-3p	Up	Acetaminophen	Krauskopf et al. 2020
**miR-19**	miR-19b-3p	Down	Acetaminophen	Vliegenthart et al. 2015
	miR-141-3p	Down	Acetaminophen	Krauskopf et al. 2017
**miR-141/200**	miR-142-3p	Down	Acetaminophen	Krauskopf et al. 2017
	miR-142-5p	Down	Acetaminophen	Krauskopf et al. 2017
	miR-200a	Down	Acetaminophen	Vliegenthart et al. 2015
	miR-204-5p	Up	HBV	Krauskopf et al. 2017
	miR-18a-3p	Down	Acetaminophen	Krauskopf et al. 2017
	miR-21*	Up	Acetaminophen	Yang X et al. 2015
	miR-21*	Up	ALF**	John et a. 2014
	miR-21-3p	Up	Acetaminophen	Krauskopf et al. 2017
	miR-21-5p	Up	Acetaminophen	Krauskopf et al. 2017
		Up	Acetaminophen	Tavabie et al. 2021
		Up	Acetaminophen	Ward et al. 2014
	miR-210-3p	Up	Acetaminophen	Krauskopf et al. 2017
	miR-217	Up	Acetaminophen	Krauskopf et al. 2020
	miR-221*	Down	Acetaminophen	Salehi et al. 2020
	miR-221-3p	Up	ALF**	John et a. 2014
		Up	Acetaminophen	Krauskopf et al. 2017
	miR-22-3p	Up	Acetaminophen	Krauskopf et al. 2020
	miR-22-5p	Up	Acetaminophen	Krauskopf et al. 2017
	miR-222-3p	Down	Acetaminophen	Krauskopf et al. 2017
**miR-23**	miR-23a*	Down	Acetaminophen	Krauskopf et al. 2015
	miR-23a-3p	Up	Acetaminophen	Krauskopf et al. 2017
		Down	Acetaminophen	Salehi et al. 2020
	miR-23b-3p	Up	Acetaminophen	Krauskopf et al. 2020
		Up	Acetaminophen	Krauskopf et al. 2017
**miR-24**	miR-24-3p	Up	Acetaminophen	Krauskopf et al. 2020
		Up	Acetaminophen	Krauskopf et al. 2017
**miR-25/92**	miR-25-3p	Up	Acetaminophen	Krauskopf et al. 2020
	miR-363	Up	Acetaminophen	Krauskopf et al. 2020
**miR-26**	miR-26b-5p	Down	Acetaminophen	Salehi et al. 2020
**miR-27**	miR-27a-3p	Up	Acetaminophen	Krauskopf et al. 2020
	miR-27b-3p	Up	Acetaminophen	Krauskopf et al. 2020
	miR-27b-3p	Up	Acetaminophen	Krauskopf et al. 2017
	miR-28-3p	Up	HBV	Krauskopf et al. 2017
**miR-28/151**	miR-28-3p	Up	Acetaminophen	Ward et al. 2014
	151a-5p	Up	Acetaminophen	Ward et al. 2014
	miR-151b-5p	Up	Acetaminophen	Ward et al. 2014
		Up	Acetaminophen	Krauskopf et al. 2017
	miR-298	Up	Acetaminophen	Krauskopf et al. 2020
**miR-29**	miR-29b*	Up	Acetaminophen	Krauskopf et al. 2020
	miR-29b*	Up	Acetaminophen	Salehi et al. 2020
	miR-29c*	Up	Acetaminophen	Tavabie et al. 2021

	miR-29c-3p	Up	Acetaminophen	Krauskopf et al. 2017
	miR-301b-3p	Up	Acetaminophen	Krauskopf et al. 2017
	miR-30a*	Down	Acetaminophen	Salehi et al. 2020
**miR-30**	miR-30a-5p	Up	Acetaminophen	Krauskopf et al. 2017
		Up	Acetaminophen	Tavabie et al. 2021
	miR-30b-5p	Down	Acetaminophen	Krauskopf et al. 2017
	miR-30c-5p	Down	Acetaminophen	Krauskopf et al. 2017
	miR-30d-5p	Up	Acetaminophen	Krauskopf et al. 2020
		Up	Acetaminophen	Yang X et al. 2015
	miR-30e-5p	Up	Acetaminophen	Krauskopf et al. 2020
	miR-3187-3p	Up	Acetaminophen	Krauskopf et al. 2020
**miR-320**	miR-320a	Down	Acetaminophen	Vliegenthart et al. 2015
		Up	Acetaminophen	Krauskopf et al. 2017
		Up	Acetaminophen	Yu et al. 2017
	miR-320b	Up	Acetaminophen	Krauskopf et al. 2017
		Up	Acetaminophen	Yu et al. 2017
	miR-320c	Up	Acetaminophen	Krauskopf et al. 2017
		Up	Acetaminophen	Yu et al. 2017
	miR-320d	Up	Acetaminophen	Krauskopf et al. 2017
		Up	Acetaminophen	Yu et al. 2017
	miR-322	Down	Acetaminophen	Krauskopf et al. 2020
	miR-328-3p	Down	Acetaminophen	Krauskopf et al. 2017
	miR-33b-5p	Up	Acetaminophen	Krauskopf et al. 2017
	miR-335-3p	Up	Acetaminophen	Krauskopf et al. 2017
	miR-335-5p	Up	HBV	Wen et al. 2018
	miR-337	Up	Acetaminophen	Krauskopf et al. 2020
**miR-34/449**	miR-34a-5p	Up	Acetaminophen	Krauskopf et al. 2020
		Up	Acetaminophen	Krauskopf et al. 2017
	miR-342-3p	Up	Acetaminophen	Krauskopf et al. 2020
		Down	Acetaminophen	Krauskopf et al. 2017
	miR-3591-5p	Up	Acetaminophen	Krauskopf et al. 2017
	miR-361-5p	Up	Acetaminophen	Krauskopf et al. 2017
	miR-3615	Down	Acetaminophen	Vliegenthart et al. 2015
	miR-362-3p	Up	Acetaminophen	Krauskopf et al. 2017
	miR-365a-3p	Up	HBV	Krauskopf et al. 2017
	miR-365b-3p	Up	HBV	Krauskopf et al. 2017
	miR-375	Up	Acetaminophen	Krauskopf et al. 2020
		Up	Acetaminophen	Yang X et al. 2015
	miR-376c	Up	Acetaminophen	Krauskopf et al. 2020
**miR-376**	miR-378a-3p	Up	Acetaminophen	Krauskopf et al. 2017
		Up	HBV	Krauskopf et al. 2017
	miR-378a-5p	Up	Acetaminophen	Krauskopf et al. 2017
	miR-378c	Up	Acetaminophen	Krauskopf et al. 2017
	miR-378d	Up	Acetaminophen	Krauskopf et al. 2017
	miR-378i	Up	Acetaminophen	Krauskopf et al. 2017
**miR-379**	miR-380	Up	Acetaminophen	Krauskopf et al. 2020
	miR-423-5p	Up	Acetaminophen	Krauskopf et al. 2020
	miR-425-5p	Up	Acetaminophen	Yang X et al. 2015
	miR-452-5p	Up	Acetaminophen	Krauskopf et al. 2017
	miR-455-3p	Up	HBV	Krauskopf et al. 2017
	miR-455-5p	Up	Acetaminophen	Krauskopf et al. 2017
		Up	HBV	Krauskopf et al. 2017
	miR-4492	Up	Acetaminophen	Krauskopf et al. 2020

	miR-4516	Up	Acetaminophen	Krauskopf et al. 2020
	miR-4732-5p	Up	Acetaminophen	Krauskopf et al. 2020
	miR-4792	Up	Acetaminophen	Krauskopf et al. 2017
	miR-483-5p	Up	Acetaminophen	Krauskopf et al. 2017
	miR-483*	Up	Acetaminophen	Yang X et al. 2015
	miR-483-5p	Up	Acetaminophen	Krauskopf et al. 2015
	miR-484	Up	Acetaminophen	Krauskopf et al. 2020
	miR-497-5p	Up	Acetaminophen	Krauskopf et al. 2017
	miR-499a-5p	Up	Acetaminophen	Krauskopf et al. 2017
	miR-505-3p	Up	Acetaminophen	Krauskopf et al. 2017
	miR-542-3p	Up	Acetaminophen	Krauskopf et al. 2020
	miR-574-3p	Down	Acetaminophen	Krauskopf et al. 2017
	miR-574-5p	Up	Acetaminophen	Krauskopf et al. 2017
	miR-584-5p	Up	Acetaminophen	Yang X et al. 2015
	miR-589-5p	Up	Acetaminophen	Krauskopf et al. 2017
	miR-592	Up	Acetaminophen	Krauskopf et al. 2017
	miR-625-3p	Down	Acetaminophen	Krauskopf et al. 2017
	miR-627-5p	Up	Acetaminophen	Krauskopf et al. 2017
	miR-6087	Up	Acetaminophen	Krauskopf et al. 2020
		Up	Acetaminophen	Krauskopf et al. 2017
	miR-188-5p	Up	Acetaminophen	Krauskopf et al. 2017
**miR-188/532**	miR-651-5p	Up	Acetaminophen	Krauskopf et al. 2017
	miR-660-5p	Up	Acetaminophen	Krauskopf et al. 2017
		Up	Acetaminophen	Krauskopf et al. 2020
	miR-710	Up	Acetaminophen	Krauskopf et al. 2020
	miR-744-5p	Down	Acetaminophen	Krauskopf et al. 2017
	miR-766-3p	Down	Acetaminophen	Krauskopf et al. 2017
	miR-885-5p	Up	Acetaminophen	Krauskopf et al. 2017
		Up	HBV	Krauskopf et al. 2017
	miR-93*	Up	Acetaminophen	Krauskopf et al. 2020
	miR-93-5p	Down	Acetaminophen	Krauskopf et al. 2017
	miR-1260a	Down	Acetaminophen	Krauskopf et al. 2017
	miR-1260b	Down	Acetaminophen	Krauskopf et al. 2017
	miR-1273h-3p	Down	Acetaminophen	Krauskopf et al. 2017
	miR-1285-5p	Up	Acetaminophen	Krauskopf et al. 2017
	miR-1290	Up	Acetaminophen	Krauskopf et al. 2017
	miR-1291	Up	Acetaminophen	Krauskopf et al. 2017
	miR-1303	Up	Acetaminophen	Krauskopf et al. 2017
	miR-1304-3p	Down	Acetaminophen	Krauskopf et al. 2017
	miR-1306-5p	Down	Acetaminophen	Krauskopf et al. 2017
	miR-1307-5p	Up	Acetaminophen	Krauskopf et al. 2017
	miR-1343-3p	Down	Acetaminophen	Krauskopf et al. 2017
	miR-1468-5p	Up	Acetaminophen	Krauskopf et al. 2017
	miR-1468-5p	Up	HBV	Krauskopf et al. 2017
	miR-4433b-5p	Down	Acetaminophen	Krauskopf et al. 2017
	miR-4448	Up	Acetaminophen	Krauskopf et al. 2017
	miR-4492	Up	Acetaminophen	Krauskopf et al. 2017
	miR-4507	Up	Acetaminophen	Krauskopf et al. 2017
	miR-4516	Up	Acetaminophen	Krauskopf et al. 2017
	miR-5096	Up	Acetaminophen	Krauskopf et al. 2017
	miR-5193	Down	Acetaminophen	Krauskopf et al. 2017
	miR-5588-5p	Up	Acetaminophen	Krauskopf et al. 2017
	miR-6741-3p	Down	Acetaminophen	Krauskopf et al. 2017
	miR-6815-5p	Up	Acetaminophen	Krauskopf et al. 2017

	miR-6852-5p	Down	Acetaminophen	Krauskopf et al. 2017
	miR-6881-3p	Down	HBV	Krauskopf et al. 2017
	miR-7704	Up	Acetaminophen	Krauskopf et al. 2017
	miR-7977	Down	Acetaminophen	Krauskopf et al. 2017

* MiR name without a tag indicating from which double-stranded RNA the described sequence comes from.

** Patients characteristics are described in table 2.
