# Supplementary material for: Insight into microRNAs-Mediated Communication between Liver and Brain: A Possible Approach for Understanding Acute Liver Failure?
Source: Int J Mol Sci. 2021 Dec 25;23(1):224. doi: 10.3390/ijms23010224 (PMC8745738; doi:10.3390/ijms23010224)
Supplement: Supplementary file 1 [file ijms-23-00224-s001.zip › Table S2.pdf]

Table S2. List of differently expressed MiRs in liver and urine of ALF patients.

MiR family	MiR	Direction of change	Patients characteristic	Specimen	Reference
	miR-122-5p	Down	ALF**	Liver	Chowdhary et al. 2017
	miR-122-3p	Down	HBV	Liver	Pan et al. 2019
	miR-1224	Up	ALF**	Liver	Roy et al. 2017
		Up	HBV	Liver	Pan et al. 2019
miR-10/100 miR-99	miR-125b-2	Down	HBV	Liver	Diaz et al. 2015
	miR-125b-2-3p	Down	HBV	Liver	Pan et al. 2019
	miR-125b-5p	Up	HBV	Liver	Singh et al. 2018
	miR-143*	Down	HBV	Liver	Diaz et al. 2015
miR-148/152	miR-148a-3p	Down	HBV	Liver	Diaz et al. 2015
	miR-150	Down	HBV	Liver	Diaz et al. 2015
	miR-150-5p	Up	HBV	Liver	Pan et al. 2019
miR-154	miR-154	Down	HBV	Liver	Diaz et al. 2015
	miR-155*	Down	HBV	Liver	Diaz et al. 2015
	miR-155-5p	Up	HBV	Liver	Pan et al. 2019
miR-15/16	miR-15a	Down	HBV	Liver	Diaz et al. 2015
miR-17	miR-18a	Down	HBV	Liver	Diaz et al. 2015
	miR-182-5p	Up	HBV	Liver	Pan et al. 2019
	miR-193b-3p	Down	HBV	Liver	Pan et al. 2019
	miR-193b-5p	Down	HBV	Liver	Pan et al. 2019
	miR-194-3p	Down	HBV	Liver	Pan et al. 2019
	miR-194-5p	Down	HBV	Liver	Pan et al. 2019
miR-200/215	miR-192*	Down	HBV	Liver	Diaz et al. 2015
	miR-192-3p	Down	HBV	Liver	Pan et al. 2019
	miR-192-5p	Down	HBV	Liver	Pan et al. 2019
miR141/200	miR-200a	Down	HBV	Liver	Diaz et al. 2015
	miR-200b-3p	Up	HBV	Liver	Pan et al. 2019
	miR-212-3p	Up	HBV	Liver	Pan et al. 2019
	miR-21-3p	Up	HBV	Liver	Pan et al. 2019
	miR-21-5p	Up	HBV	Liver	Pan et al. 2019
	miR-223-3p	Up	HBV	Liver	Pan et al. 2019
	miR-24-2-5p	Up	HBV	Liver	Pan et al. 2019
miR-302	miR-302a*	Up	Acetaminophen	Urine	Yang X et al. 2015
	miR-30a-5p	Down	HBV	Liver	Diaz et al. 2015
	miR-30c-1	Down	HBV	Liver	Diaz et al. 2015
	miR-30e	Down	HBV	Liver	Diaz et al. 2015
	miR-30b-3p	Down	HBV	Liver	Pan et al. 2019
	miR-3175	Up	HBV	Liver	Pan et al. 2019
	miR-330-3p	Up	HBV	Liver	Pan et al. 2019
	miR-342-5p	Up	HBV	Liver	Pan et al. 2019
	miR-370-3p	Up	HBV	Liver	Pan et al. 2019
	miR-375	Up	Acetaminophen	Urine	Yang X et al. 2015
	miR-375	Down	HBV	Liver	Pan et al. 2019
	miR-376c-3p	Up	HBV	Liver	Pan et al. 2019
	miR-382-5p	Up	HBV	Liver	Pan et al. 2019
	miR-387a	Down	HBV	Liver	Diaz et al. 2015
	miR-409-3p	Up	HBV	Liver	Pan et al. 2019
	miR-421	Up	HBV	Liver	Pan et al. 2019
	miR-432-5p	Up	HBV	Liver	Pan et al. 2019
	miR-433-3p	Up	HBV	Liver	Pan et al. 2019
	miR-483-5p	Down	HBV	Liver	Pan et al. 2019
	miR-503-5p	Up	HBV	Liver	Pan et al. 2019

	miR-542-5p	Down	HBV	Liver	Diaz et al. 2015
	miR-574-3p	Down	HBV	Liver	Pan et al. 2019
	miR-625*	Down	HBV	Liver	Diaz et al. 2015
	miR-625-5p	Up	HBV	Liver	Pan et al. 2019
	miR-629*	Down	HBV	Liver	Diaz et al. 2015
	miR-629-5p	Up	HBV	Liver	Pan et al. 2019
	miR-650	Up	HBV	Liver	Pan et al. 2019
	miR-885-3p	Down	HBV	Liver	Pan et al. 2019
	miR-885-5p	Down	HBV	Liver	Pan et al. 2019
	miR-940	Up	Acetaminophen	Urine	Yang X et al. 2015
	miR-9-3p	Up	Acetaminophen	Urine	Yang X et al. 2015

* MiR name without a tag indicating from which double-stranded RNA the described sequence comes from.

** Patients characteristics are described in table 2.
